# Systems analysis of quantitative shRNA-library screens identifies regulators of cell adhesion

**DOI:** 10.1186/1752-0509-2-49

**Published:** 2008-06-13

**Authors:** XiaoDong Huang, Jean YJ Wang, Xin Lu

**Affiliations:** 1 Division of Biological Sciences, University of California at San Diego, La Jolla, CA 92093, USA; 2 Moores Cancer Center, University of California at San Diego, La Jolla, CA 92093, USA; 3 Division of Hematology/Oncology, Department of Medicine, School of Medicine, University of California at San Diego, La Jolla, CA 92093, USA; 4 Department of Family and Preventive Medicine, University of California at San Diego, La Jolla, CA 92093, USA; 5 Genentech Inc., 1 DNA Way, South San Francisco, CA 94080, USA; 6 Abbott Laboratories, 100 Abbott Park Rd, North Chicago, IL 60063, USA

## Abstract

**Background:**

High throughput screens with RNA interference technology enable loss-of-function analyses of gene activities in mammalian cells. While the construction of genome-scale shRNA libraries has been successful, results of large-scale screening of those libraries can be difficult to analyze because of the relatively high noise levels and the fact that not all shRNAs in a library are equally effective in silencing gene expression.

**Results:**

We have screened a library consisting of 43,828 shRNAs directed against 8,500 human genes for functions that are necessary in cell detachment induced by a constitutively activated c-Abl tyrosine kinase. To deal with the issues of noise and uncertainty of knockdown efficiencies, we employed an analytical strategy that combines quantitative data analysis with biological knowledge, i.e. Gene Ontology and pathway information, to increase the power of the RNAi screening technique. Using this strategy we found 16 candidate genes to be involved in Abl-induced disruption of cell adhesion, and verified that the knockdown of IL6ST is associated with enhanced cell attachment.

**Conclusion:**

Our results suggest that the power of genome-wide quantitative shRNA screens can be significantly increased when analyzed using a systems biology-based approach to identify functional gene networks.

## Background

RNA interference with small interfering RNAs (siRNA) and short hairpin RNAs (shRNA) has proven to be a powerful tool for functional genetic studies, especially for human cells where application of classical genetic tools is limited [1]. Short double-stranded RNAs between 19–29 base pairs can efficiently silence gene expression by mediating the sequence-specific degradation of target mRNAs [2] and leading to suppression of endogenous gene expression [3]. With whole genome sequences available for humans and many other model organisms, it is now possible to use shRNA libraries to perform genome-wide screens that examine the contribution of every gene to a specific biological process, by creating RNA interfering libraries to perturb the function of all known genes [4-10]. Among the current methods for large-scale shRNA library screens, one of the most efficient techniques uses lentivirus to transfer the whole pooled shRNA library to the cell population of interest where the shRNA sequences are selected from the probes used by commercial microarray product such as the Affymetrix arrays, and then evaluates the relative abundance of the shRNAs within cells before and after treatment by oligo-microarray.

However, there are some obstacles that must be overcome in order to extract reliable data from such a screening experiment. For instance, although some shRNAs have been reported to down-regulate genes efficiently and specifically, there is currently no large-scale shRNA library that has been thoroughly validated to be specific for the genes targeted. In addition, it is possible that some of the shRNAs might be lost in the study or simply be lethal to the cells, which would preclude them from providing useful data. Furthermore, the shRNAs recovered from a given population of cells must be amplified before being measured by microarray and it is likely that this amplification step will not be uniform across replicates. Finally, the commercial microarrays used to analyze shRNA abundance in these screens were originally designed to measure large mRNA transcripts rather than shorter shRNA molecules. The ability to extract statistically significant results from these microarrays came in part from the fact that most of these arrays were designed using multiple short oligo probes to measure one mRNA. However, the size of the shRNA limits the readout for any given shRNA to only a single probe, reducing the statistical power of this method. While the inclusion of multiple shRNAs for each gene of interest helps to address this problem, this is still limited by the efficacy of each of the individual shRNAs. Some shRNA libraries are designed to have gene-specific barcodes that can be detected by microarrays. Although the separate design of RNAi sequence and barcode sequence can decouple the requirement for RNAi sequence and array hybridization sequence, it also doubles the effort to design and synthesize the library, and the barcodes are still short oligos detected by single probes which inevitably will result in a higher noise level than multiple probe detections of mRNAs. Collectively, these obstacles make the signal-to-noise ratio in current shRNA screening experiments very low compared to traditional microarray studies.

To facilitate a reliable result from this type of experiments, we developed an analytical strategy that takes advantage of the Gene Ontology (GO) information. GO terms are a summary of biological information about genes. It was organized into a hierarchy under one of the three root categories: Biological Process (BP), Cellular Component (CC), and Molecular Functions (MF). Each of these three categories gives rise to a hierarchy of subgroups in which the biological process or function that the genes contribute to becomes more specific. Several GO based mRNA expression analysis methods have been proposed to group genes into functional categories and conduct statistical inference on sets of genes sharing similar functions rather than on the individual genes themselves [11,12]. These methods can significantly decrease the noise level in the data thus increase the statistical power. RNAi screening data generally have an even higher noise level than those from mRNA expression arrays, but in current shRNA screening researches no such GO based method has been reported. Therefore we developed a rank-based Gene Ontology method to identify GO terms that are significantly associated with the enrichment or depletion of shRNA in a screening study. In addition to the increased statistical power by grouping shRNAs according to the functional category of their target genes, this method also drastically reduces the problem of off-target effects, as it is unlikely that multiple shRNAs have a similar off-target effect in a specific GO group. Since network analysis has been shown to be a powerful tool to understand biological responses by providing a global view of gene products and their relationships [13], we also conducted a pathway analysis based on gene interaction information retrieved from literatures.

In this study, we applied these two bioinformatics methods discussed above to analyze the relative abundance data from an shRNA library screen for genes involved in the regulation of cell adhesion. The screen was based on an experimental system where induced expression of a constitutively active Abl tyrosine kinase leads to cell detachment. The *c-Abl *gene encodes a ubiquitously expressed non-receptor tyrosine kinase that undergoes nucleocytoplasmic shuttling [14]. The nuclear Abl is activated by DNA damage to stimulate apoptosis [15-18]. The cytoplasmic Abl is activated by growth factors and cell adhesion to regulate F-actin dynamics associated with cell spreading, cell migration and neurite outgrowth [19]. Interestingly, we have found that the induction of the constitutively activated Abl kinase leads to cell detachment (Huang et al., Submitted). As resistance to detachment represents a simple phenotypic selection criterion, we screened an shRNA library targeting 8,500 human genes for shRNAs that were either enriched or depleted in cells that remained attached despite the expression of activated Abl kinase. Using the analysis strategy discussed above, we constructed a gene network for the regulation of cell adhesion and found several major hub genes in this network. As a proof of principle, we have experimentally confirmed one of the putative effectors, the membrane protein IL6ST as being required for cell detachment. Taken together, these results support the strategy of using systems biology approach to extract meaningful results from shRNA screening studies.

## Results

### shRNA library screening of AblPP-induced HEK293 cell detachment

The c-Abl tyrosine kinase is held in an inactive conformation in the absence of activation signals [20-22]. This inactive conformation can be disrupted by substituting two proline residues for two glutamate residues in the linker region between the SH2 and catalytic domains (P242E/P249E), leading to constitutive kinase activity [23]. We placed this AblPP mutant gene under the control of TET-regulated promoter (TET-on system) and stably transfected this construct into HEK293 cells (referred to AblPP cells from this point on). Upon AblPP expression, around 80% of the host cells detached from the supporting matrix in about 2 hours (Huang, X., et al., submitted).

**Figure 1:**
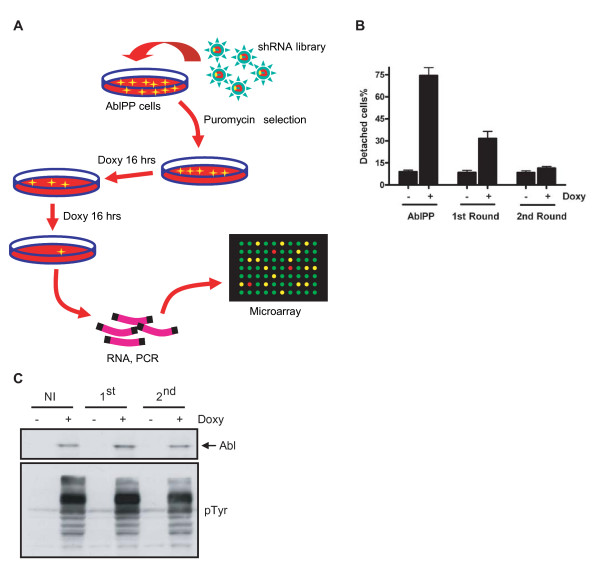
**shRNA library screening of Abl kinase-induced cell detachment**. (A) Schematic outline of the experimental setup for the identification of effectors in AblPP-induced cell detachment. Details provided in Methods. (B) Percentage of detached cells after each round of AblPP induction. AblPP cells before (NI) and after (1^st ^and 2^nd^) shRNA library transduction was subjected to AblPP-induced cell detachment. Following the 2^nd ^round of AblPP induction, AblPP cells did not detach anymore. (C) AblPP was induced to similar levels and exhibited similar kinase activity during each round of AblPP induction.

We infected the AblPP cells with an 8.5 K human GeneNet™ shRNA library, which contains 43,828 shRNAs targeting 8,500 human genes (Fig. 1A), with each gene being targeted by 4–5 shRNAs. The shRNAs in this library were designed based on the Affymetrix Human Genome Focus Array and therefore the relative abundance of each shRNA can be measured by the intensity of the signal from the corresponding probe on this array. AblPP cells infected with this library were first selected with puromycin to eliminate uninfected cells. This procedure also eliminated shRNAs that interfere with cell growth. The stably transduced cells were then subjected to two rounds of AblPP induction and cells that remained attached after the second round of AblPP expression were collected (Fig. 1B). The resistance of these cells to AblPP-induced detachment was neither due to a loss of AblPP expression nor a loss of AblPP kinase activity (Fig. 1C). We then PCR amplified the shRNA sequences from these attached cells according to the instructions of the GeneNet™ manual, hybridized the recovered sequences to the appropriate Affymetrix microarrays and measured the abundance of the shRNA sequences. We performed 3 biological repeats, hybridizing shRNA sequences recovered from the library-transduced cells prior to AblPP expression as well as those recovered from the twice-selected attached cells. The raw data from the six microarrays are provided in [see Additional file 1].

### Rank-based Gene Ontology analysis

The shRNA abundance data collected before and after selection for attached cells were quantile normalized and log transformed. The histogram of shRNA abundance showed a heavy tail distribution (data not shown), therefore we took a log transform to stabilize the variation for better power in later statistical analysis. The histogram of log transformed shRNA abundance prior to selection also showed that many shRNAs were of low abundance even before AblPP was induced [see Additional file 2]. Some of these shRNAs might have been eliminated during expansion of the culture since they knocked-down the essential genes for cell growth. To filter out these low-abundance shRNAs, we fitted the data from the non-selected cells with a Gaussian Mixture model and selected 13,140 shRNAs that were highly abundant in all 3 biological replicates (with posterior probability above 0.99) for further analysis.

Because the abundance of each shRNA was measured by a single probe instead of using multiple ones as in many mRNA expression arrays, the noise level in the data was relatively high and classical microarray analysis methods such as t-statistics followed by FDR were not suitable to analyze this type of data. For example, we applied paired t-statistics to compare the hybridization intensity for each of the 13,140 shRNAs before and after selection and corrected the p-values by the FDR [24] and the Q-value method [25,26]. With this analysis, no shRNA with FDR corrected p-value below 0.05 could be found, and the minimum FDR corrected p-value was 0.184. The Q-value method returned an error message because the distribution of p-value was deviant from that usually observed in an mRNA expression array. Clearly, the relatively high noise level in the shRNA abundance data precluded it from being analyzed with typical statistical methods based on individual genes.

To extract information efficiently from the shRNA abundance data, we designed a rank-based Gene Ontology analysis method. Our method was similar to the Gene Set Enrichment Analysis (GSEA) method [11] in that it also used Gene Ontology information to group shRNAs based on the known functions of their target genes. But instead of using t-statistics, we used the rank of the log ratios of these shRNA-target genes to improve the robustness of the analysis. We then calculated the mean of the ranks of genes that belong to a GO term instead of the weighted Kolmogorov-Smirnov test in GSEA method [11] and compared to the mean rank in permutations for the significance (See Methods for details). This rank-based ontology analysis could also be applied to other ontology schemes, such as the Panther ontology [27,28] and KEGG [29], where genes are grouped based on their similarities by prior biological knowledge, but the results will depend on the ontology assignment of genes, and the structure of the ontology used. Although Gene Ontology might be inaccurate or partially accurate in some cases, it is still the most used ontology scheme, especially in annotating commercial microarrays and other high-throughput data. Since the enrichment and depletion of shRNA have entirely opposite biological meaning and there were multiple shRNA probes per each target gene in our screening data, we ranked genes and selected GO terms according to their degree of enrichment and depletion separately (see Methods for details). By analyzing the collective data for a group of genes, rather than any single gene alone, we effectively increased the statistical power of our analysis. As a result, we were able to find groups of genes significantly enriched or depleted in cells that remained attached despite the induction of AblPP.

With criterion of a p-value below 0.01, we identified 7 GO terms in the category of Biological Process (BP), 2 GO terms in the category of Cellular Component (CC) and 9 GO terms in the category of Molecular Function (MF) whose constituent genes were significantly enriched after selection. We also selected 9 GO terms in BP, 2 GO terms in CC, and 9 GO terms in MF whose genes were significantly depleted after selection. These GO terms are listed in Table 1 together with their p-values. Among these GO terms, some are known to be related to cell adhesion, such as transmembrane receptor protein tyrosine kinase signaling pathway (BP), negative regulation of cell proliferation (BP), extracellular matrix (CC), and collagen binding (MF).

**Table 1:** Significantly enriched or depleted GO terms.

Enriched GO terms			
Category	GO.ID	GO.Term	P value

BP	7169	transmembrane receptor protein tyrosine kinase signaling pathway	0.000108
	6069	ethanol oxidation	0.004402
	30183	B cell differentiation	0.004668
	8285	negative regulation of cell proliferation	0.004690
	6641	triacylglycerol metabolism	0.005847
	42592	Homeostasis	0.009063
	6631	fatty acid metabolism	0.009442

CC	118	histone deacetylase complex	0.001873
	5578	extracellular matrix (sensu Metazoa)	0.008284

MF	16566	specific transcriptional repressor activity	0.001047
	15248	sterol transporter activity	0.004570
	4024	alcohol dehydrogenase activity, zinc-dependent	0.004699
	4407	histone deacetylase activity	0.006790
	4300	enoyl-CoA hydratase activity	0.008276
	3700	transcription factor activity	0.008297
	8484	sulfuric ester hydrolase activity	0.008613
	5518	collagen binding	0.009071
	4924	oncostatin-M receptor activity	0.009582

Depleted GO terms			

Category	GO.ID	GO.Term	P value

BP	6959	humoral immune response	0.000405
	6356	regulation of transcription from RNA polymerase I promoter	0.002046
	15986	ATP synthesis coupled proton transport	0.004346
	1701	embryonic development (sensu Mammalia)	0.004439
	30187	melatonin biosynthesis	0.006552
	6906	vesicle fusion	0.007231
	7286	spermatid development	0.008267
	31424	Keratinization	0.008692
	17148	negative regulation of protein biosynthesis	0.009817

CC	16469.00	proton-transporting two-sector ATPase complex	0.002401
	19898.00	extrinsic to membrane	0.006794

MF	16165	lipoxygenase activity	0.000954
	4111	creatine kinase activity	0.003959
	46933	hydrogen-transporting ATP synthase activity, rotational mechanism	0.004516
	46961	hydrogen-transporting ATPase activity, rotational mechanism	0.004516
	4983	neuropeptide Y receptor activity	0.004746
	8048	calcium sensitive guanylate cyclase activator activity	0.005498
	17096	acetylserotonin O-methyltransferase activity	0.006552
	4857	enzyme inhibitor activity	0.007804
	15232	heme transporter activity	0.009458

### Pathway analysis

**Figure 2:**
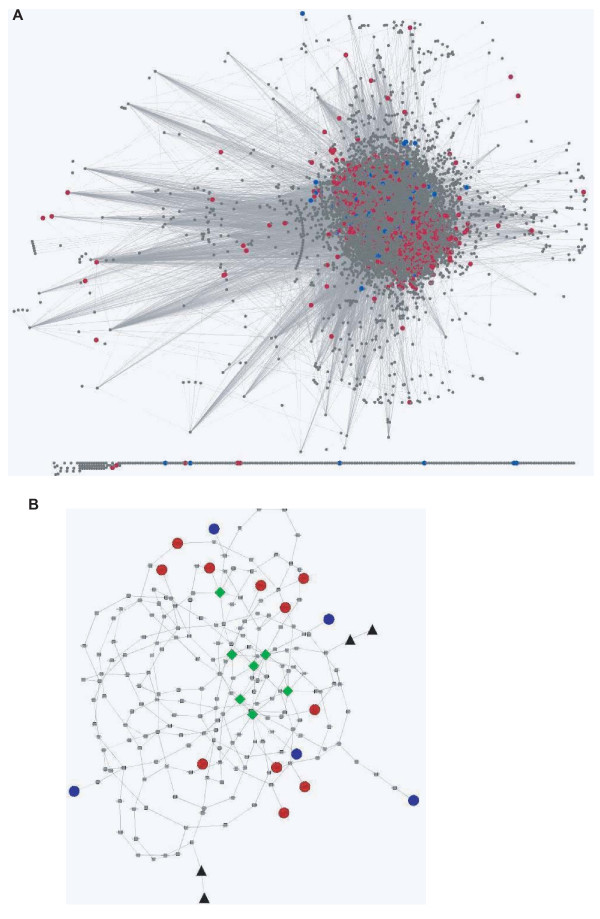
**The human gene interaction network and the shortest path network connecting the 16 significantly enriched or depleted target genes**. (A) Human gene interaction network. Nodes represent genes and lines connecting nodes represent gene interactions. Red and blue spots high-lighted represent genes belong to significantly enriched or depleted GO terms with a p-value of rank sum statistics below 0.01. (B) The shortest path network connecting all 16 significantly enriched or depleted genes. Red and blue spots represent genes significantly enriched or depleted. Green diamonds represent hub genes with connectivity greater than 5. Black triangles represent pathways connecting Abl and ROCK1 into this network.

Among the 8,500 genes targeted by the shRNA library, a total of 833 genes belonged to at least one of the significantly enriched or depleted GO terms [see Additional file 3]. We built a human protein interaction network based on the information queried from the Biomolecular Object Network Database (BOND) and the Human Protein Reference Database (HPRD), and mapped the selected genes into this network (Fig. 2A). The BOND and HPRD databases are collections of literature reported gene interactions discovered by a variety of techniques, which are the most comprehensive collection of gene interaction knowledge currently available [30]. Since our shRNA library covers barely one third of all human genes and some of the shRNAs might not be effective or might have off-target effects, a pathway analysis based on the complete gene interaction network can gain us a more global picture of the inter-relationship of the selected genes. For the enriched and depleted genes, we used paired t-statistics to compare their relative abundance before and after selection. There were 14 genes significantly enriched and 7 genes significantly depleted with p-values below 0.01 (Table 2 and 3), out of which 11 enriched and 5 depleted genes were also in the gene interaction network. We then tried to find the shortest paths to connect each pair of the 16 genes to construct a Shortest Path Network (SPN) (Fig. 2B). From this SPN, we further identified 7 hub genes with connectivity greater than 5 (Table 4) [31,32]. In an independent candidate-approach study, we have identified ROCK1 kinase to be required for AblPP-induced cell detachment (Huang X., et al., submitted). Therefore we also integrated c-Abl and ROCK1 into this shortest path network. The pathways we found were listed in Additional file 4.

**Table 2:** Target genes that shRNA were significantly enriched.

**Gene**	**P-value**	**T-statistics**	**Entrez Gene**	**Significant GO term**	**GO category**	**shRNA sequence**
EDN2	0.0011	20.9528	1907	transmembrane receptor protein tyrosine kinase signaling pathway	BP	5'AGCTCTGCTGGAAGA ACTGCATGGGGA3'
ARNTL2	0.0020	15.7851	56938	transcription factor activity	MF	5'TTACCTTGTAGCTCT CAATCATCAGAA3'
ZNF232	0.0030	12.7514	7775	transcription factor activity	MF	5'TATAACTCACATCT TGTTGTCCACCAG3'
ADH5	0.0040	11.1071	128	ethanol oxidation	BP	5'GTATAACCTAAACC ATCTACTCTTTAG3'
COL15A1	0.0043	10.7380	1306	extracellular matrix (sensu Metazoa)	CC	5'GCAACTGCTGTTCGT ACACAGAAACAG3'
EMP3	0.0071	8.2852	2014	negative regulation of cell proliferation	BP	5'TGAATCTCTGGTACG ACTGCACGTGGA3'
MYT2	0.0072	8.2309	8827	transcription factor activity	MF	5'AAGCAGATGATATT CCAGACGGTATTA3'
ZNF219	0.0079	7.8689	51222	transcription factor activity	MF	5'GGTAGTGGGCCCTCA GGGGCGATTAGC3'
IL6ST	0.0080	7.8232	3572	oncostatin-M receptor activity	MF	5'TGTCCAGTATTCTAC CGTGGTACACAG3'
MEOX2	0.0080	7.8013	4223	transcription factor activity	MF	5'GCACTCACAATGACA ACCAGAGCCAGT3'
EPHB4	0.0092	7.2683	2050	transmembrane receptor protein tyrosine kinase signaling pathway	BP	5'TTCTACCGTCCTTGT CATAACTTTGTG3'
KLF10	0.0093	7.2104	7071	negative regulation of cell proliferation; transcription factor activity	BP/MF	5'ATTGGGTGTAGATTT CTGACATCAAAA3'
TFEC	0.0097	7.0622	22797	transcription factor activity	MF	5'GCTATGCAATTATGC TCTGTGTTTCAT3'
NPC1	0.0098	7.0363	4864	sterol transporter activity	MF	5'GATGCTCTCATAAGG CCCAGGAAGGAT3'

**Table 3:** Target genes that shRNA were significantly depleted.

**Gene**	**P-value**	**T-statistics**	**Entrez Gene**	**Significant GO term**	**GO category**	**shRNA sequence**
ABCB7	0.0019	-16.2715	22	heme transporter activity	MF	5'GAATGCCACATGGATATGACACCCAAG3'
RFXANK	0.0027	-13.5118	8625	humoral immune response	BP	5'GGAGAGATTGAGACCGTTCGCTTCCTG3'
CD28	0.0031	-12.5570	940	humoral immune response	BP	5'GAACTGTTGGATTTACCCTGGCACGTG3'
ASMT	0.0035	-11.8293	438	melatonin biosynthesis; acetylserotonin O-methyltransferase activity	BP/MF	5'GCATGACTGGCCAGACGACAAAGTCCA3'
PPYR1	0.0039	-11.2743	5540	neuropeptide Y receptor activity	MF	5'GCATCCATTTGCATCGTGAAGACTGGC3'
CKB	0.0053	-9.6779	1152	creatine kinase activity	MF	5'GCTGCGGGCAGGTGTCGATATCAAGCT3'
SPOCK3	0.0066	-8.6420	50859	enzyme inhibitor activity	MF	5'TAGTGCTTGGGATCGTACATGTTAATT3'

**Table 4:** The hub genes identified from the shortest path network connecting significantly enriched or depleted shRNA target genes

Entrez Gene ID	Gene
22981	*RP4-691N24.1*
4188	*MDFI*
2130	*EWSR1*
57473	*GM632*
509	ATP5C1 *
4110	*MAGEA11*
60	*ACTB*
6310	*ATXN1*
4088	*SMAD3*
5764	*PTN*

*The gene ATP5C1 belong to depleted GO terms, but was not significantly depleted in the study (p-value = 0.21).

### IL6ST contributes to AblPP-induced cell detachment

**Figure 3:**
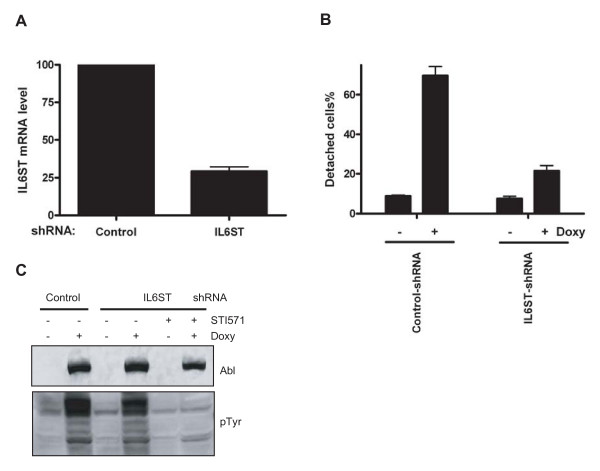
**IL6ST rescued AblPP-induced cell de-adhesion**. (A) Relative abundance of IL6ST mRNA level in AblPP cells infected by IL6ST shRNA or control shRNA was measured by quantitative PCR. The mRNA level from AblPP cells infected by control shRNA was set as 100. (B) AblPP was induced in AblPP cells infected by IL6ST shRNA or control shRNA by doxycycline addition and cell detachment assay was measured at 4 hours. (C) The knockdown of IL6ST did not affect the expression of AblPP or its kinase activity.

Based on the pathway analysis of the shRNA screening results, we decided to validate the IL6ST out of the candidate genes, because it has been shown to regulate cell-cell adhesion in cultured cardiomyocytes [33]. To validate the function of IL6ST in our study, we used new shRNA sequences and infected AblPP cells with a lentiviral vector to express these shRNAs (see Methods for details). Quantitative PCR results indicated that the mRNA level of IL6ST was significantly reduced (Fig. 3A). The cells depleted of IL6ST were resistant to AblPP-induced cell detachment (Fig. 3B), and this phenotype was not due to either a loss of AblPP expression or a loss of phosphorylation in AblPP cells (Fig. 3C), suggesting that IL6ST is a *bona fide *effector in Abl kinase-induced cell detachment pathway.

## Conclusion

In this study, we combined quantitative shRNA screening with the current literature knowledge, including Gene Ontology and gene-interaction networks, to identify pathways that are associated with the regulation of AblPP-induced cell detachment. By grouping genes based on their GO categories and conducting statistical analysis of sets of genes, we show that the experimental noise associated with quantitative shRNA screening can be effectively reduced to allow identification of biologically relevant shRNA gene targets. By mapping the identified genes onto the protein interaction network and performing topological analyses of the network architecture, we discovered not only genes that are significantly modulated, but also how these genes are inter-connected and how they might influence entire regulatory pathways. The shRNA library we used in this study covers only about one third of entire human genome, so what we found is only part of the mechanisms that control cell adhesion. But as a proof-of-principle study, we showed the power of combining shRNA library screening with advanced statistical and systems analysis. With future version of whole genome scale shRNA libraries, we would be able to acquire more complete information with similar analytical strategies.

## Methods

### Cell culture

HEK293 cells expressing AblPP under the control of TET-regulated promoter (TET-on system) were generated as described (Huang et al., in revision). These cells were cultured in DMEM media containing 10% FBS, penicillin/streptomycin and 0.1% β-mercaptoethanol. Cells were routinely treated with 2 μg/ml doxycycline at 37°C to induce cell detachment unless noted otherwise.

### Lentivirus production and infection of AblPP cells

Lentiviral particles were generated by transient transfection of 293FT cells by IL6ST shRNAs (from Sigma) and VSV-G-expression plasmids. The supernatants were collected 48 hours after transfection. Stable cell lines expressing IL6ST shRNAs were generated by transduction with the retroviral supernatants in the presence of 8 μg/ml polybrene, with infected cells selected for resistance to puromycin (2.0 μg/ml).

We used MISSION™ TRC shRNA Target Set (TRCN0000058285) from Sigma to knockdown the endogenous IL6ST in our validation study. The functional sequence in the shRNA vector is "CCGGCCCATACTCAAGGCTACAGAACTCGAGTTCTG TAGCCTTGAGTATGGGTTTTTG" to target the *IL6ST *gene sequence (^1273^CATACTCAAGGCTACAGAACT^1293^).

### shRNA screen, provirus recovery, DNA microarray

The lentiviral shRNA library used was 8.5 K human GeneNet™ shRNA library constructed in pFIV-H1-puro vector, consisting of 43,828 shRNAs directed against 8,500 human genes. The infection of AblPP cells by this library was performed by following the standard protocol from GeneNet™ [34]. Briefly, 1 × 10^6^AblPP cells were infected with the lentiviral shRNA library at an MOI of 0.5. Twenty-four hours after infection, the cells were selected with 2 μg/ml puromycin. Selected cells were subjected to two rounds of AblPP-induced cell detachment by treatment with 2 μg/ml doxycycline for 16 hours each time. The screened cells were amplified; total RNA was extracted and reverse transcribed to cDNA. The proviral sequences in the remaining attached colonies were obtained by two rounds of PCR. In the first round of PCR, the template used was cDNA and the primers were (forward: 5'-AATGTCTTTGGATTTGGGAATCTTA-3'; reverse: 5'-AAAAGGGTGGACTGGGATGAGTA-3'). During the second round of PCR two nested primers were used (forward: 5'-ATCGTCAATCACCTTCCTGTCAGA-3', and this primer was biotin-labeled at its 5' end; reverse: 5'-ATAGAAAGAATGCTTATGGACGCTA-3'). The amplified sequences of shRNA targets were used for hybridization with Affymetrix Human Genome Focus Arrays using standard protocols. Three biological repeats were performed, for both the surviving population and control population of cells.

### Western blot

Whole cell lysates were prepared in RIPA buffer (50 mM Tris-HCl [pH 7.4], 150 mM NaCl, 1% NP-40, 0.25% sodium deoxycholate, 0.1% SDS, 0.5 mM EDTA, 1 mM EGTA, 1 mM DTT) plus protease inhibitors (from Sigma); 50–100 μg of total protein were resolved by SDS-PAGE, transferred onto PVDF membranes, blocked in 5% nonfat dry milk/TBST (20 mM Tris-HCl [pH 7.5], 150 mM NaCl, 0.05% Tween-20) and incubated with primary antibodies overnight at 4°C. Membranes were washed 3 × 10 minutes in TBST and incubated with HRP-conjugated secondary antibodies for 1–3 hours at room temperature. After 3 × 10 minutes washing, membranes were incubated with enhanced ECL reagent (from Pierce) for 1 minute and exposed to X-ray films. Anti-pTyr antibody 4G10 and anti-Abl antibody 8E9 were both prepared from our lab.

### Cell detachment assay

AblPP cells were seeded on 12-well plate coated with poly-L-lysine and cultured in 37°C for overnight. The cells were treated with 2 μg/ml doxycycline for different time and were fixed with 4% paraformaldehyde for 15 minutes, washed with PBS and observed under phase-contrast microscope. The bright and round cells were treated as positive cells. In each assay, at least 300 cells were counted.

### Data analysis

The shRNA levels of the 3 pre- and post-AblPP induction experiments were normalized by quartiles, and then log2 transformed using R language [35] and BioConductor packages [36]. In order to identify high- and low-abundant shRNAs, the shRNA levels of pre-induction were fitted by a Gaussian-Mixture model using EM algorithm and the probability of each shRNA belonging to high abundance group was calculated.

When selecting the shRNAs that were enriched after selection, we used the minimum of the log ratios among the 3 repeats to reduce the effect of noise. Then we selected one shRNA that has the maximum enrichment ratio after AblPP induction as the representative for each gene. To select shRNAs that were depleted after selection we calculated the pre-/post-ratio and repeated the above procedure. To reduce the effect of possible experimental and technique noise in the data, we used only the rank of the log ratios of genes. To calculate the p-value of genes being significantly enriched or depleted in the study, we used paired t-statistics to compare the shRNA abundance in pre- and post-induction.

### Rank sum statistics of Gene Ontology

We used Gene Ontology to group genes based on their functional category and selected significant GO terms that are associated with cell detachment. GO terms that are too general containing more than 500 genes or too special containing less than 2 genes were ignored. For the rest of the GO terms, we calculated the sum of rank of the genes that belong to each GO term, and compared it to that of 1,000 randomly selected gene sets with the same number of genes to calculate the Z-score and corresponding p-value.

### Protein interaction analysis

We constructed human protein interaction network from documented molecular interactions retrieved from Biomolecular Object Network Databank (BOND) [37] and Human Protein Reference Database (HPRD) [38] and visualized by Cytoscape software V2.4 [39]. While building the interaction network, we kept only the protein-protein interactions since the cell detachment is a quick process in our system. It is likely that this process is regulated by only protein-protein interactions without gene transcription and translation involved with the exception of *AblPP *gene. To find the shortest pathways to connect given genes in the protein interaction network, we first located the biggest connected component in the whole human protein network that contains 9,150 proteins and 35,969 interactions, and ignored the rest less connected components. Then we assigned a weight to the edges connecting these proteins by the sum of clustering coefficient of the 2 proteins each edge connects [30]. Finally, the shortest path between a pair of given proteins were found by the Dijkstra's shortest path algorithm [40].

## Authors' contributions

XH and JYJW initiated this project; XH did the biological experiments; XL developed the rank-based GO analysis method and did the pathway analysis of the candidate genes; JYJW and XL selected the candidate genes to be subject to validation; JYJW guided XH and XL on performing the biological experiments and analyzing the data; XH and XL executed the writing with input from JYJW. All authors have read and approved the final manuscript.

## Supplementary Material

Additional file 1 Supplementary Table I. Raw data of the 3 replicated shRNA screening experiment. Click here for file

Additional file 2 Supplemental Figure 1. Mixture Gaussian model of shRNA abundance. The histogram of log transformed shRNA abundance prior to selection. Click here for file

Additional file 3 Supplementary Table II. The 833 genes selected that belong to any significant enriched or depleted GO terms. Click here for file

Additional file 4 Supplementary Table III. All pathways connecting each pair of selected genes. Click here for file
