# Supplementary material for: Systems analysis of quantitative shRNA-library screens identifies regulators of cell adhesion
Source: BMC Syst Biol. 2008 Jun 13;2:49. doi: 10.1186/1752-0509-2-49 (PMC2441606; doi:10.1186/1752-0509-2-49)
Supplement: Additional file 2 — Supplemental Figure 1. Mixture Gaussian model of shRNA abundance. The histogram of log transformed shRNA abundance prior to selection. [file 1752-0509-2-49-S2.pdf]

Supplemental Figure 1

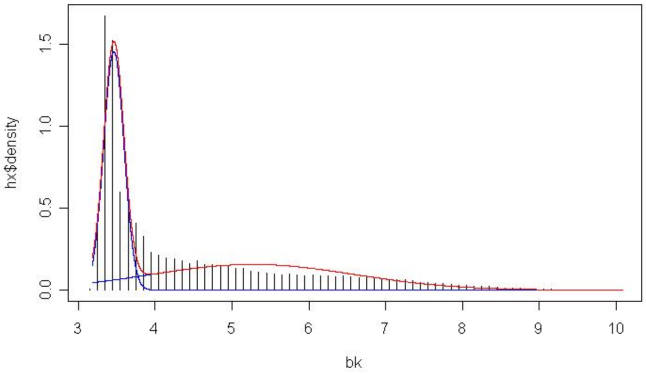

Supplemental Figure 1. Mixture Gaussian Model of shRNA abundance

X-axis represents the log of relative abundance of the shRNA in pre-treatment samples measured by Affymetrix microarray, and Y-axis represents the density of the distribution. The distribution showed that there were lots of shRNAs having very low abundance before AlbPP induction, they may be lost in the cell culture or lethal to the cell and thus can not provide useful data in the study. The overall distribution was fit by a mixture Gaussian distribution, as shown in the figure the blue curves represent the 2 Gaussian components and the red curve represents the fitted 2-component Gaussian-Mixture model.
